# On a Peculiar Affection of the Cornea in Nurses

**Published:** 1843-01

**Authors:** 


*On a peculiar affection of the Cornea in Nurses.* By Prof. Nasse.—A malignant form of keratitis or inflammation of the cornea occasionally accompanies puerperal attacks, and in general terminates fatally. This affection, however, is not of a malignant nature, and appears at any time during the whole period of nursing, from a month after delivery to a year and a half, if the child be suckled so long. The eye is felt irritable and the conjunctiva is seen injected with blood. Occasionally catarrhal symptoms attend the complaint, at other times little vesicles appear over the surface of the conjunctiva. Sometimes rheumatic symptoms are present, at other times it comes on with a vesicular cutaneous eruption over the face. The conjunctival inflammation rapidly passes to the cornea and is accompanied by the usual darting pains in the eye and margin of the orbit. From the third to the eighth day an abscess forms within the layers of the cornea, when the inflammatory symptoms diminish, and if nothing be done to put an end to the complaint, it bursts into the anterior chamber and occasions hypopion.

The disease is not peculiar to any age, constitution, or season; but is in every case preceded by great lassitude, debility, and leanness, brought on by excessive lactation, in fact, seems to be a disease of debility. Blood-letting is consequently never indicated, but blisters behind the ears, diaphoretics combined with bitter infusions, quinine and sulphuric acid, a tonic diet, and above all, the giving up suckling the child, generally effect a cure in about three weeks. It is mentioned that the separation of the child is the most important part of the treatment; and cases are related where the child being allowed to suckle before the cure was completed, brought it back with increased severity, and could not be stopped till the child was again removed, when the disease rapidly gave way.—*Edin. Med. and Surg. Journ.*, July, 1842.

